# Effect of bevacizumab on refractory meningiomas: 3D volumetric growth rate versus response assessment in neuro-oncology criteria

**DOI:** 10.1093/noajnl/vdae128

**Published:** 2024-08-13

**Authors:** Sara Faye Borenstein, Ruth Eliahou, Alexandra Amiel, Alisa Talianski, Jonathan Ofer, Shaked Even-Haim, Andrew Kanner, Yosef Laviv, Dror Limon, Tali Siegal, Shlomit Yust-Katz

**Affiliations:** Faculty of Medicine, Tel Aviv University, Tel Aviv, Israel; Department of Radiology, Rabin Medical Center – Beilinson Hospital, Petach Tikva, Israel; Faculty of Medicine, Tel Aviv University, Tel Aviv, Israel; Department of Radiology, Rabin Medical Center – Beilinson Hospital, Petach Tikva, Israel; Faculty of Medicine, Tel Aviv University, Tel Aviv, Israel; Department of Neuro-Oncology, Sheba Medical Center, Tel HaShomer, Israel; Faculty of Medicine, Tel Aviv University, Tel Aviv, Israel; Department of Neurosurgery, Rabin Medical Center – Beilinson Hospital, Petach Tikva, Israel; Faculty of Medicine, Tel Aviv University, Tel Aviv, Israel; Department of Neurosurgery, Rabin Medical Center – Beilinson Hospital, Petach Tikva, Israel; Faculty of Medicine, Tel Aviv University, Tel Aviv, Israel; Faculty of Medicine, Tel Aviv University, Tel Aviv, Israel; Brain Tumor Center, Davidoff Cancer Center, Rabin Medical Center – Beilinson Hospital, Petach Tikva, Israel; Radiation Oncology Unit, Davidoff Cancer Center, Rabin Medical Center – Beilinson Hospital, Petach Tikva, Israel; Faculty of Medicine, Tel Aviv University, Tel Aviv, Israel; Faculty of Medicine, Tel Aviv University, Tel Aviv, Israel

**Keywords:** 3DVGR, bevacizumab, RANO criteria, refractory meningiomas

## Abstract

**Background:**

Meningiomas are the most common primary tumor in the central nervous system. About 15%–20% are aggressive and tend to recur and progress despite conventional treatment. Bevacizumab has been found to be effective in the treatment of refractory meningiomas in retrospective studies. The Response Assessment in Neuro-Oncology (RANO) criteria are widely used to assess the effect of treatment. Recent studies suggest that the 3D volumetric growth rate (3DVGR) may be more accurate for irregularly shaped tumors. The aim of this study was to compare these approaches.

**Methods:**

Twenty patients with refractory meningiomas were treated with bevacizumab. Tumors were measured using the RANO criteria and 3DVGR before and after initiation of treatment by 2 radiologists using PACS and BRAIN LAB iPLAN software, respectively, findings were compared.

**Results:**

A total of 46 lesions were included in the final analysis. Bevacizumab was shown to be effective by both assessment methods. According to RANO criteria, the rate of progression-free survival at 6 months was 47%. According to 3DVGR, all lesions were characterized by either a decrease in volume or stable growth after treatment initiation. A decrease in 3DVGR of 50% or more was found in 90% of lesions. In several patients, there were discordances between RANO criteria and 3DVGR.

**Conclusions:**

Although RANO criteria are widely accepted for evaluation of response to treatment of meningiomas, 3DVGR seems to generate more precise measurements of irregularly shaped tumors. The results of this study offer important evidence that bevacizumab may be beneficial in treating refractory meningiomas.

## Lay Summary

Meningiomas are the most common brain tumor. Some meningiomas are aggressive and do not respond well to standard treatments. In order to see whether a meningioma is responding to treatment, doctors usually compare the size of tumors in 2-dimensions using MRIs before and after treatment. The authors of this study wanted to see whether using 3-dimensional measurements would provide any benefit over 2D measurements. To do this, the authors treated 20 patients with aggressive meningiomas using a drug called bevacizumab and compared the tumor sizes before and after treatment using MRIs. They found that treatment with bevacizumab was effective when using both 2D and 3D measurements. Six months after treatment, 47% of patients had stable tumors using 2D measurements, while 90% of tumors had either shrunk or stopped growing using 3D measurements.

**Key Points**

- Both the RANO criteria and the 3DGVR reliably assess response to treatment of meningiomas.
- The 3DVGR method appears to generate more precise measurements in irregularly shaped meningioma.
- Bevacizumab is a promising treatment for aggressive refractory meningiomas.

**Importance of the Study**

The standard method for monitoring the effect of treatment in meningioma is the Response Assessment in Neuro-Oncology (RANO) criteria. Recent studies suggest that 3D volumetric growth rate (3DVGR) may be more accurate for irregularly shaped tumors. In this study, both methods were used to monitor the effect of bevacizumab in 20 patients with 46 aggressive meningiomas. Compared to the RANO criteria, 3DVGR seemed to generate more precise measurements in cases of irregularly shaped meningiomas. This finding may imply that the benefit shown for a potential treatment might depend on the method used to monitor response. Overall, bevacizumab showed promise as an effective treatment for aggressive refractory meningioma.

Meningioma is the most prevalent primary central nervous system (CNS) tumor.^1^ The WHO classification of meningiomas is based on pathological and molecular features as the radiological appearance is frequently nonspecific.^2,3^ About 80% of meningiomas are classified CNS WHO grade 1, and the remainder present with aggressive characteristics and are classified CNS WHO grades 2 (atypical) or 3 (anaplastic). The conventional treatment for aggressive meningiomas includes surgical resection and adjuvant radiation therapy. In many cases, the disease progresses despite treatment.^4,5^

Retrospective studies of the efficacy of different conventional systemic treatments reported progression-free survival rates at 6 months (PFS6) of 26%–29%.^6^ An exception was bevacizumab, a vascular endothelial growth factor (VEGF) inhibitor, which showed both clinical and radiographic benefits in several clinical studies.^7–12^ The most recent prospective study reported a PFS6 of up to 90% for grade 1 refractory meningiomas and 66% for grade 2/3 meningiomas using bevacizumab therapy.^12^

There was no consensus regarding the clinical and radiographic endpoints for meningioma clinical trials until 2019, when the response assessment in neuro-oncology (RANO) criteria were established,^13^ significantly improving uniformity in practice. However, the RANO criteria are based on 2-dimensional measurements which do not fully portray the true size of the lesion. This limitation is accentuated in cases of atypical, irregularly shaped meningiomas. More importantly, the RANO criteria are not sensitive to small size variations in the tumor. Meningiomas have a slower growth rate than gliomas, so longer surveillance is required to evaluate treatment response with the RANO criteria.^13^

The accuracy of volumetric tumor measurements has improved tremendously in recent years. Studies that included patients with meningioma showed that the 3-dimensional volumetric growth rate (3DVGR) was a more accurate measure for treatment assessment than the RANO criteria.^14–17^

The aim of the present study was to compare the effectiveness of the RANO criteria and the 3DVGR for the assessment of response to bevacizumab treatment in patients with refractory meningioma.

## Material and Methods

This retrospective study was approved by the Institutional Review Boards of Rabin Medical Center (RMC-05010-23) and Sheba Medical Center (5889-19) in Israel.

### Patient Selection

The study cohort included adult patients with aggressive meningiomas who failed conventional treatment and were treated with bevacizumab (from 2018 to 2022). Aggressive meningioma was defined as a refractory grade 2 or 3 tumor or a grade 1 tumor that behaved aggressively and did not respond to conventional treatment with surgery and radiation. Only patients in whom 2 sequential head magnetic resonance imaging (MRI) studies were acquired prior to initiation of bevacizumab therapy and at least one examination was performed within 3 months after initiation of bevacizumab treatment—were enrolled in the study.

### Clinical Data

For each patient, the following data were retrieved from the medical files: sex, age, WHO grade (according to WHO 2016^2^), predisposing radiation, number of prior surgeries and radiation therapies, prior chemotherapy, number of bevacizumab cycles and dose, Karnofsky Performance Scale (KPS) score and steroid dosage before and after therapy (T1 time point).

### Image Acquisition and Analysis

Radiological and clinical evaluations were conducted at 3-time points using the RANO criteria and 3DVGR: Before initiation of treatment (baseline, by 2 MRI scans), 3 months after treatment initiation (T1), and at 6 to 9 months after treatment initiation (T2).

MRI was performed in all cases using 3T scanners (Siemens Healthcare Magnetom Vida and Tesla Philips Ingenia). The studies were reviewed by 2 neuroradiologists who evaluated the scans separately. Measurements were obtained on 3D T1-weighted acquisitions (slice thickness 1.0 mm) after gadolinium injection.

The 2 longest orthogonal lengths, as indicated by the RANO criteria, were reviewed using PACS. The RANO criteria were applied for each patient at each time point. Each lesion underwent observational assessment for intensity of enhancement which was graded on a scale of 0 (none) to 3 (avidly enhancing).^14^ The enhancing pattern and composition of the lesion (solid/semisolid/necrotic) were documented as well.

The volume of each lesion was determined using BRAIN LAB iPlan, version 4.1.2. The pretreatment volumetric growth rate was calculated for each lesion using the equation described by Graillon et al.^17^:


$$\begin{array}{l} TGRpre∼{-}_{\;ttt}=100\times \left\{{\left(V_{0}/V_{-t}\right)}^{1/t}-1\right\}, \end{array}$$

where TGRpre~− _ttt_ is the tumor growth rate prior to initiation of treatment (in percentage), V_−t_ and V_0_ are the pretreatment volumes (in cubic centimeters), and *t* is the time in months between the 2 examinations. The post-treatment volumetric growth rate was extrapolated in relation to the interval between measurements using the equation:


$$\begin{array}{lll} V_{t}=\; & V_{0}\times exp(TGR_{t<3}\times t\times \left\{t<3\right\}\; & +{TGR}_{t^{3}3}\times \left(t-3\right)\times I\left\{t^{3}3\right\}), \end{array}$$

where TGR_t<3_ is the tumor growth rate in the first 3 months and TGR_t≥3_ is the tumor growth after 3 months, {∙} is the indicator function (which equals 1 when the condition is verified, and 0 if the condition is not verified), and V_0_ and V_t_ are the tumor volumes (in cubic centimeters) on the date of initiation of treatment and after t months, respectively. Each lesion response to treatment was categorized according to the proposed pattern response classification as described by Graillon et al.^17^

Since most patients had more than one lesion, an average growth rate of all lesions per patient was calculated.

A decrease in tumor volume growth rate of 50% or more was considered a major decrease in growth rate, as defined in a recent study of patients with refractory meningiomas treated with everolimus and octreotide.^18^

### Statistical Analysis

Fisher’s exact test was used to compare contingency outcomes of improvement, steroids down tapering, and KPS score, between patterns. Wilcoxon signed-rank test was applied to compare paired nonparametric data sets. Hazard ratios and 95% CI were calculated for PFS using the Kaplan–Meier plots and Log-rank test. A *P* value < .05 was considered significant. Statistical analysis was performed using GraphPad Prism, version 10.10 for Windows (GraphPad Software, La Jolla, CA, USA; www.graphpad.com).

## Results

### Patient Characteristics

Of 39 patients screened for the study, 20 (13 male, 7 female) fulfilled the inclusion criteria. Mean patient age was 67.9 years. The median number of radiation pretreatments was 2, and the median number of surgeries was 3. The characteristics of the patients are presented in Table 1.

**Table 1.** Patients’ Characteristics of 20 Patients With Refractory Meningiomas

Characteristics	Number
Age (yr), mean (range)	67.9 (42-87)
Sex, (F/M), n	7/13
*Childhood radiotherapy, n (%)*	
Yes	8 (40%)
No	12 (60%)
*No. of lesions, n (%)*	
Single	8 (40%)
Multiple (>1)	12 (60%)
No. of previous surgeries, median (range)	3 (0-7)
No. of previous radiation treatments, median (range)	2 (1-10)
*Previous chemotherapy* ^*^ *, n (%)*	
Yes	7 (35%)
No	13 (65%)
*WHO grade, n (%)*	
1	1 (5%)
2	15 (75%)
3	3 (15%)
Unknown	1 (5%)
KPS pretreatment, mean (range)	50% (20–90)
Dose of dexamethasone pretreatment (mg), mean (range)	2.66 (0–8)
No. of bevacizumab treatments, median (range)	21 (6–54)

WHO, World Health Organization, KPS, Karnofsky Performance Status (score).

^*^Keytruda, Olaparib, Somatostatin analogs, mTOR inhibitors.

### Lesion Characteristics

The 20 patients had a total of 46 lesions. Five patients, with a total of 14 lesions, did not undergo follow-up imaging at the T2 time point (2 patients died, 2 were lost to follow-up and one was forced to discontinue due to healing hindering of a wound). The 2 neuroradiologists who evaluated the scans separately showed high agreement in their measurements. Duration time between MRI studies is further summarized in Supplementary Table 1. During follow-up, the mean enhancing score decreased, the percentage of solid lesions decreased, and the percentage of necrotic lesions increased (Table 2). An example of enhancement response is illustrated in Supplementary Figure 1.

**Table 2.** Average Enhancing Score and Lesion Characteristics at Each Evaluation

	Average enhancing score	Lesion characteristics		
		Solid (%)	Semisolid^*^ (%)	Necrotic (%)
Baseline (T0): treatment initiation	2.67	58.7	34.8	6.5
T1: 3 months from treatment initiation	1.87^†^	32.6	34.8	32.6
T2: 3 to 9 months after treatment initiation	1.54^†^	28.0	37.5	34.4

^*^Lesion with mixed solid and necrotic areas.

^†^Wilcoxon signed-rank test showed a significant difference in average enhancing score between T0 and T1 (*P* < .05) and between T0 and T2 *(P* < .05).

### Response to Bevacizumab According to the RANO Criteria

According to the RANO criteria, all 20 patients had progressive disease (PD) prior to treatment. At T1, there was an improvement in the RANO categorization in 12 patients (60%): to stable disease in 9 (45%), to minor response in 2 (10%), and to partial response in 1 (5%). There was no change in the remaining 8 patients (40%) as they were still categorized as PD despite treatment.

Follow-up data from T2 were available for 15 patients (75% of the initial cohort- per protocol). At this time point, the response was assessed in relation to baseline or nadir, as elaborated in the report from the RANO Working Group.^13^ The final category was stable disease in 10 patients (66.6%) and PD in 5 (33.3%).

**Figure 1.**
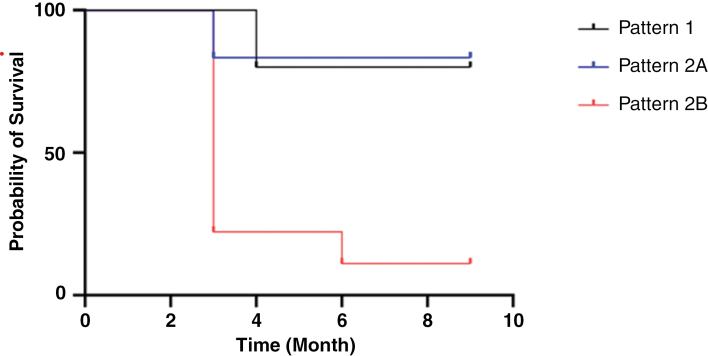
Progression free survival per patient according to Graillon’s proposed pattern of response to treatment during the surveillance time.

According to RANO criteria, the overall PFS6 rate was 47%. While using the patterns of response proposed by Graillon et al, figure 1 illustrates an advantage patterns 1 and 2A have compared to pattern 2B in PFS. This was also statistically significant when using Kaplan–Meier log rank test.

### Clinical Outcome

Bevacizumab treatment was tolerated well with no reported adverse effects directly attributed to treatment. One patient mentioned above, developed an independent wound in his scalp that hindered full recovery due to anti-VEGF activity the treatment holds. Clinical improvement was documented in 55% of the patients. The steroid dose was reduced in 89% of patients. While using the patterns of response proposed by Graillon et al, there was no significant correlation between the pattern categorization and steroid dose change nor in KPS variations as a result of treatment.

### Response to Bevacizumab According to the 3DVGR

**Figure 2.**
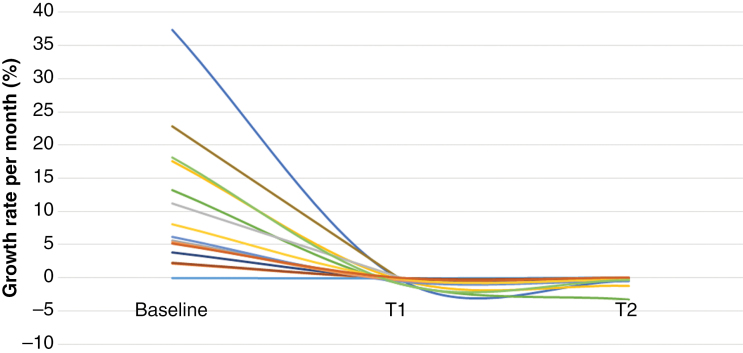
Effect of bevacizumab treatment on the growth rate of refractory meningioma per patient (only those who had 2 sequential studies after treatment initiation). Each patient is represented by a different color. Each line represents the mean growth rate of that patient’s lesions.

Appreciating the overall change in 3DVGR per patient over the course of the study is displayed in Figure 2. In the first 3 months after initiation of bevacizumab treatment, there was a sharp decrease in the 3DVGR, which reached a plateau at 3–9 months after treatment initiation.

The percentage of change in the 3DVGR that may be considered clinically significant is debatable. However, a recent study that included patients with refractory meningiomas treated with everolimus and octreotide set a 50% decrease in volume growth rate as the appropriate threshold defining a major change.^18^ The growth rate of the present cohort at different intervals from treatment initiation is described in Table 3. A decrease of more than 50% in 3DVGR compared to baseline was noted in 82.6% of lesions (38/46) at T1 and 90% of lesions (27/30) at T2. Wilcoxon signed-rank test yielded a significant difference in 3DGVR between baseline and T1 (*P* < .05), and between baseline and T2 (*P* < .05). Further details of each lesion characteristics are presented in Supplementary Table 2.

**Table 3.** Characteristics of the Different Patterns of Response in Each Time Points Per Lesion

	Pattern 1					Pattern 2A					Pattern 2B				
	*N*	Mean	SD	Median	*P* value	*N*	Mean	SD	Median	*P* value	*N*	Mean	SD	Median	*P* value
Baseline (T0) GR	19	13.03	9.83	6.45		13	10.42	10.64	3.51		14	25.18	30.75	6.2	
T1 GR	19	−0.55	0.24	−0.425	<.0001^†^	13	−0.09	0.318	−0.07	.0005^†^	14	−0.05	0.235	−0.08	.0001^†^
T2 GR	13	−0.96	0.69	−0.45	.0002^†^	8	−0.74	1.123	−0.045	.0156^†^	11	−0.05	0.2	−0.05	.001^†^

GR, growth rate; N, number of lesions; SD, standard deviation.

^†^Wilcoxon signed-rank test showed a significant difference in mean GR between T0 and T1 (*P* < .05) and between T0 and T2 (*P* < .05) in each pattern group.

Although there is no independent validation of the mathematical model derived from a small cohort study, we used the response patterns of classification proposed by Graillon et al to analyze or results at T1 and T2 time points.^17^

We noticed 3 patterns of response: pattern 1 (decrease in volume) was found in 41.3 % of the lesions. Pattern 2A (stabilization of volume) was found in 28.26% of lesions (13/46); pattern 2B (slight increase in volume with GR slow down) was found in 30.43% of lesions (14/46). None of the lesions were categorized as pattern 3-(persistent increase in volume). We found a significant difference in GR at T1 and T2 in comparison to baseline GR consistently throughout the different patterns when using a Wilcoxon signed-rank test (Table 3).

Out of the 15 patients who had 2 sequential studies after initiation of treatment (T1 and T2), we noticed a change in the pattern of growth only in 18.75% of the lesions (6/32): 3 lesions categorized as 2A progressed to 2B, while 2 lesions categorized as 2B improved to 1 and 2A and 1 lesion from 2A improved to 1.

Eight patients at T1 and 4 patients at T2 were categorized PD according to the RANO classification despite a negative 3DVGR average per patient (Table 4). According to Graillon’s classification, of these, 6 lesions were categorized as pattern 1, 7 as 2A, and 12 as pattern 2B out of 46 lesions at T1. At the T2 time point, 4 lesions were classified as pattern 1, 4 as 2A and 5 as pattern 2B out of 32 lesions.

**Table 4.** RANO Versus Growth Rate (Measurement Per Patient) and 3DVGR Pattern Classification (Measured Per Lesion)

	T0			T1			T2		
	RANO	3DVGR	3DVGR patterns	RANO	3DVGR	3DVGR pattern	RANO	3DVGR	3DVGR pattern
PD	20 (100%)	17.76% (3.6, 44.3)	NA	8 (40%)	−0.16 % (−0.29, −0.04)	1- 6/46	4 (26.6%)	−0.44 % (−1.2, −0.02)	1- 4/32
						2A- 7/46			2A- 2/32
						2B-12/46			2B- 5/32
SD				9 (45%)	−0.17 % (−0.04, −0.34)	1- 7/46	11 % (73.4%)	−0.48 % (−3.2, 0.04)	1- 11/32
						2A- 6/46			2A- 3/32
						2B- 2/46			2B- 7/32
MR				2 (10%)	−0.9 % (−0.9, −0.9)	1- 5/46			
						2A- 0/46			
						2B- 0/46			
PR				1 (5%)	−0.6 %	1- 1/46			
						2A- 0/46			
						2B- 0/46			
CR									

Values are presented as mean 3DVGR (%) and (range).

The mean growth rate of all lesions in a single patient was calculated.

3DVGR patterns according to Graillon’s proposed response classification to treatment listing number of lesions (1- decrease in volume; 2A- volume stabilization; 2B- minor volume increase with growth rate decrease).

T0, pretreatment time point; T1, 3 months from treatment initiation; T2, 3 to 9 months after treatment initiation RANO, Response Assessment in Neuro-Oncology [criteria]; 3DVGR, 3-dimensional volumetric growth rate; PD, progressive disease; SD, stable disease; MR, minor response; PR, partial response; CR, complete response; NA, not applicable.

## Discussion

This study assessed the response to bevacizumab in patients with refractory meningiomas using both the conventional RANO criteria and 3DVGR. According to both methods, bevacizumab was an effective treatment. When the RANO criteria were applied, PFS6 was 47% compared to 26%–29% for conventional therapy as documented in previous studies.^12,13^ Our rate is lower than reported in the most recent prospective study of refractory meningiomas in which the PFS6 was up to 90% for grade 1 and 66% for grade 2/3.^12^ The difference may be explained by the lower mean KPS score in our cohort relative to the earlier one or due to inherent bias of retrospective studies. According to the 3DVGR, all lesions showed a decrease in size or stabilization in growth rate after treatment initiation, and more than 80% regressed by at least 50%.

There is an inherent challenge to comparing categorical (RANO) and numerical (3DVGR) variables in this context. Nevertheless, we could easily observe that several lesions showed a considerable decrease in growth rate (also according to Graillon’s proposed Response Pattern Classification) even though the patients were categorized PD according to the RANO criteria. There are several possible reasons for the differences in response measured by the 2 methods. First, the RANO criteria are based on 2D measurements and are not sensitive to slow changes. Second, individuals with multiple lesions will be categorized as PD according to the RANO criteria if only one lesion is growing, even if the remainder shrinks or stabilizes. With the 3DVGR approach, each lesion is measured independently. However, there is no standard method to assess an individual with multiple lesions.^15–18^ In these cases, in the present study, we calculated the mean growth rate of all lesions in a single patient. Third, if a tumor continues to grow but at a slower rate, the patient may still be categorized PD by the RANO criteria.

Therefore, when a new drug or treatment regimen is assessed with the well-accepted RANO criteria, the response may be underappreciated. At the same time, although 3DVGR is a very sensitive measure, no standard cutoff for significant change has been established. Several studies suggested a threshold of a 40%–50% decrease or more in volumetric growth rate as clinically significant.^18,19^

In the past decade, the volumetric growth rate has become recognized as a more accurate measure for the assessment of response to new treatments in oncology.^19,20^ More and more studies have been using this approach in addition to the conventional criteria, as the 3DVGR is based solely on the radiological response, whereas the RANO criteria incorporate also clinical measures.

VEGF has been shown to play a significant role in neovascularization, tumor growth, and genesis of edema in meningiomas.^12^ Bevacizumab acts by inhibiting the vascular endothelial growth rate, which is directly correlated with enhancement.^9^ Accordingly, in our study, bevacizumab had a significant effect on the enhancement of the lesions and changed their characteristics, as indicated by the increase in the number of lesions that became necrotic during treatment. It is noteworthy that besides affecting volume shrinkage and a reduction in edema, as expected from its mechanism of action, bevacizumab also induced a significant and continuous reduction in tumor growth.

The limitations of this study are the relatively small cohort and the retrospective design. Also, our study was inspired by Grallion’s mathematical model that was based on a small cohort of patients (*n* = 32) and was not independently validated.^17^ Therefore, the validity of our findings is limited.

## Conclusion

Although the RANO criteria are widely accepted for treatment response evaluation, 3DVGR seems to be more precise for meningiomas that are irregularly shaped and that progress slowly with relatively small inter-scan variations. The 3DVGR method also makes it possible for clinicians to detect subtle therapeutic effects that might be clinically meaningful. These findings may imply that the benefit shown for a potential treatment might depend on the method used to monitor response. The results of this study offer important evidence that bevacizumab may be beneficial in treating refractory meningiomas, yet further prospective studies need to be conducted.

## Supplementary Material

vdae128_suppl_Supplementary_Tables

vdae128_suppl_Supplementary_Figure
