# Correction to: Effect of bevacizumab on refractory meningiomas: 3D volumetric growth rate versus response assessment in neuro-oncology criteria

**DOI:** 10.1093/noajnl/vdae164

**Published:** 2024-10-04

This is a correction to: Sara Faye Borenstein *et al.*, Effect of bevacizumab on refractory meningiomas: 3D volumetric growth rate versus response assessment in neuro-oncology criteria, *Neuro-Oncology Advances*, Volume 6, Issue 1, January-December 2024, vdae128, https://doi.org/10.1093/noajnl/vdae128

The second author’s name has been corrected.

