# Supplementary material for: Effect of bevacizumab on refractory meningiomas: 3D volumetric growth rate versus response assessment in neuro-oncology criteria
Source: Neurooncol Adv. 2024 Aug 13;6(1):vdae128. doi: 10.1093/noajnl/vdae128 (PMC11520742; doi:10.1093/noajnl/vdae128)
Supplement: vdae128_suppl_Supplementary_Tables [file vdae128_suppl_Supplementary_Tables.docx]

| Patient # | Interval 1 | Interval 2 | Interval 3 |
| --- | --- | --- | --- |
| \| 1 \| \| --- \| \| 2 \| \| 3 \| \| 4 \| \| 5 \| \| 6 \| \| 7 \| \| 8 \| \| 9 \| \| 10 \| \| 11 \| \| 12 \| \| 13 \| \| 14 \| \| 15 \| \| 16 \| \| 17 \| \| 18 \| \| 19 \| \| 20 \| | \| 3 \| \| --- \| \| 2.5 \| \| 2 \| \| 5 \| \| 2 \| \| 3 \| \| 5.5 \| \| 5.5 \| \| 3 \| \| 2 \| \| 3 \| \| 1.5 \| \| 5 \| \| 2 \| \| 1.5 \| \| 3 \| \| 2 \| \| 1 \| \| 2.5 \| \| 14 \| | \| 3 \| \| --- \| \| 2.5 \| \| 3 \| \| 4 \| \| 3 \| \| 3 \| \| 5.5 \| \| 4.5 \| \| 3.5 \| \| 9 \| \| 4 \| \| 2 \| \| 2.5 \| \| 4 \| \| 5 \| \| 4 \| \| 3 \| \| 2.5 \| \| 3 \| \| 5 \| | \| 4 \| \| --- \| \| 2 \| \| 2.5 \| \| 2 \| \| 2.5 \| \| 2 \| \| 5 \| \| 2.5 \| \| NA \| \| 6 \| \| NA \| \| NA \| \| 3 \| \| 3 \| \| 3.5 \| \| 1.5 \| \| NA \| \| 2 \| \| NA \| \| 3 \| |

Table 1s. Intervals between MRI tests per patient

Interval 1- Number of months between MRI 1 and MRI 2 before initiation of treatment.

Interval 2- Number of months between MRI 2 before treatment and MRI post treatment (T1 time point).

Interval 3- Number of months between first MRI post treatment (T1 time point) and second MRI post treatment (T2 time point).

| Lesion # | Patient # | WHO Grade | Age | Gender | 3DVGR pre-treatment (%/month) | Response Pattern #* |  |
| --- | --- | --- | --- | --- | --- | --- | --- |
| 1  2  3  4  5  6  7  8  9  10  11  12  13  14  15  16  17  18  19  20  21  22  23  24  25  26  27  28  29  30  31  32  33  34  35  36  37  38  39  40  41  42  43  44  45  46 | | 1  1  1  1  2  3  3  4  4  4  5  6  6  7  8  8  9  9  9  9  10  10  11  11  11  12  12  13  14  15  15  15  16  16  16  17  17  17  18  18  18  19  19  20  20  20 | 3  3  3  3  2  2  2  2  2  2  2  1  1  No Data  2  2  2  2  2  2  2  2  2  2  2  3  3  2  2  2  2  2  2  2  2  2  2  2  2  2  2  2  2  3  3  3 | 70  70  70  70  57  58  58  71  71  71  52  66  66  81  87  87  63  63  63  63  42  42  71  71  71  66  66  71  74  63  63  63  84  84  84  75  75  75  69  69  69  70  70  68  68  68 | M  M  M  M  F  M  M  M  M  M  F  F  F  M  M  M  M  M  M  M  M  M  M  M  M  M  M  M  M  M  M  M  F  F  F  M  M  M  F  F  F  F  F  F  F  F | 24.57  20.68  7.53  96.68  2.19  4.86  6.45  17.21  5.56  29.82  0  14.9  11.51  3.85  1.16  3.314  3.51  0  5.4  5.4  21.11  24.5  0.15  83.03  47.8  24.75  63.78  6.12  5.41  6.2  25.6  1.64  11.67  7.63  4.78  0  0  17.87  0  0  54.49  0  14.28  1.53  12.23  1.77 | 1- Decrease  1- Decrease  1- Decrease  2B- Slight Increase  2A- Stabilization  1- Decrease  1- Decrease  2A- Stabilization  2B- Slight Increase  2A- Stabilization  2A- Stabilization  1- Decrease  1- Decrease  2A- Stabilization  2B- Slight Increase  2B- Slight Increase  2A- Stabilization  1- Decrease  1- Decrease  2B- Slight Increase  2B- Slight Increase  2B- Slight Increase  2A- Stabilization  2B- Slight Increase  2B- Slight Increase  2A- Stabilization  1- Decrease  1- Decrease  1- Decrease  2B- Slight Increase  2B- Slight Increase  2B- Slight Increase  2A- Stabilization  1- Decrease  2B- Slight Increase  1- Decrease  2A- Stabilization  1- Decrease  1- Decrease  1- Decrease  1- Decrease  1- Decrease  2A- Stabilization  2A- Stabilization  2B- Slight Increase  2A- Stabilization |

Table 2s. Lesion Characteristics

*Patterns according to Graillon et al proposed classification.

**Legends to supplementary Figure**

**Fig-s. 1** An illustrated example of the change in enhancement in a patient with multiple meningiomas during treatment with bevacizumab. A- before initiation of treatment- graded as solid- enhancing score of 3, B- T1 time point- semi solid with enhancing score of 2 and C-T2 time point- semi solid with enhancing score of 1.
