# Supplementary figures and images for: Effect of bevacizumab on refractory meningiomas: 3D volumetric growth rate versus response assessment in neuro-oncology criteria
Source: Neurooncol Adv. 2024 Aug 13;6(1):vdae128. doi: 10.1093/noajnl/vdae128 (PMC11520742; doi:10.1093/noajnl/vdae128)

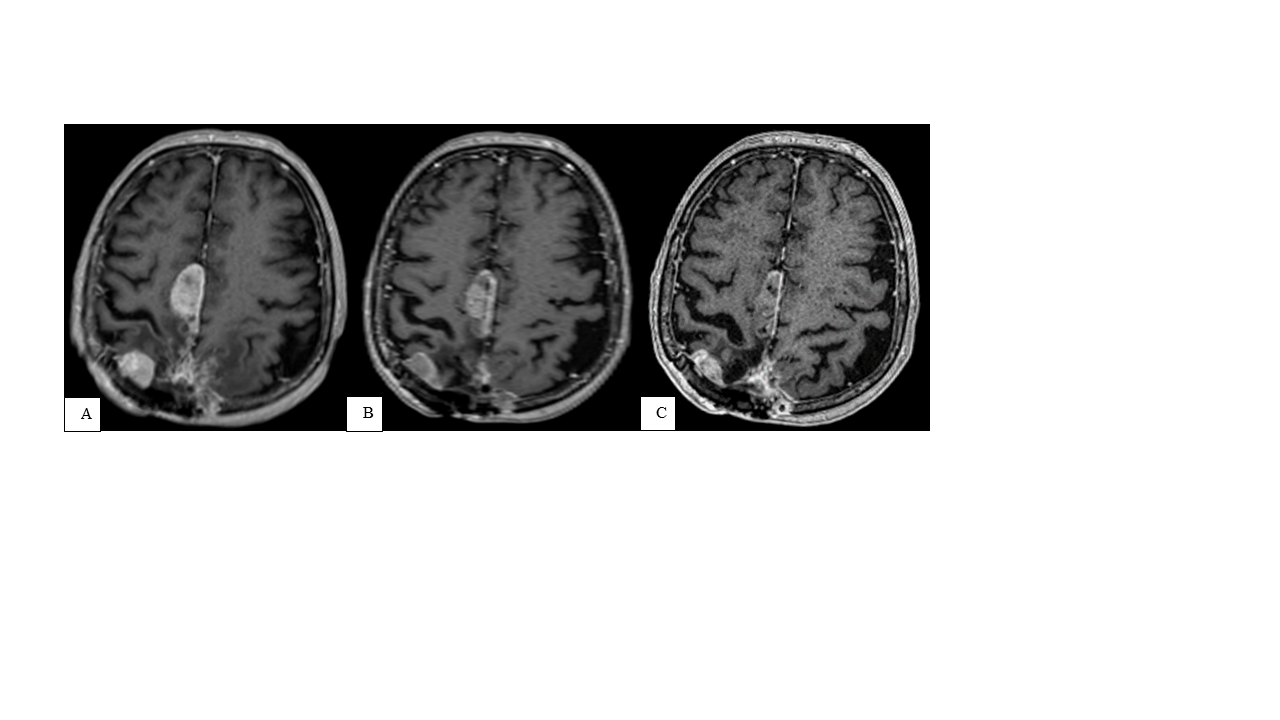

Supplement: vdae128_suppl_Supplementary_Figure [file vdae128_suppl_Supplementary_Figure.tif]
